# Evaluating an intervention to improve the safety and experience of transitions from hospital to home for older people (*Your Care Needs You*): a protocol for a cluster randomised controlled trial and process evaluation

**DOI:** 10.1186/s13063-023-07716-z

**Published:** 2023-10-14

**Authors:** Rebecca Lawton, Jenni Murray, Ruth Baxter, Gerry Richardson, Sarah Cockayne, Kalpita Baird, Laura Mandefield, Stephen Brealey, Jane O’Hara, Robbie Foy, Laura Sheard, Alison Cracknell, Edmund Breckin, Catherine Hewitt, on behalf of the PACT research team

**Affiliations:** 1 Yorkshire Quality and Safety Research group, Bradford Institute for Health Research, Bradford, UK; 2 School of Psychology, University of Leeds, Leeds, UK; 3 Centre for Health Economics, University of York, York, UK; 4 York Trials Unit, University of York, York, UK; 5 School of Healthcare, University of Leeds, Leeds, UK; 6 Leeds Institute of Health Sciences, University of Leeds, Leeds, UK; 7 Leeds Centre for Older People’s Medicine, Leeds Teaching Hospitals NHS Trust, Leeds, UK

**Keywords:** Transitions of care, Discharge, Cluster randomised controlled trial, Hybrid interventions, Patient involvement, Older people, Study protocol, Readmission

## Abstract

**Background:**

Older patients often experience safety issues when transitioning from hospital to home. The ‘Your Care Needs You’ (YCNY) intervention aims to support older people to ‘know more’ and ‘do more’ whilst in hospital so that they are better prepared for managing at home.

**Methods:**

A multi-centre cluster randomised controlled trial (cRCT) will evaluate the effectiveness and cost-effectiveness of the YCNY intervention.

Forty acute hospital wards (clusters) in England from varying medical specialities will be randomised to deliver YCNY or care-as-usual on a 1:1 basis. The primary outcome will be unplanned hospital readmission rates within 30 days of discharge. This will be extracted from routinely collected data of at least 5440 patients (aged 75 years and older) discharged to their own homes during the 4- to 5-month YCNY intervention period. A nested cohort of up to 1000 patients will be recruited to the study to collect secondary outcomes via follow-up questionnaires at 5-, 30- and 90-day post-discharge. These will include measures of patient experience of transitions, patient-reported safety events, quality of life and healthcare resource use. Unplanned hospital readmission rates at 60 and 90 days of discharge will be collected from routine data.

A process evaluation (primarily interviews and observations with patients, carers and staff) will be conducted to understand the implementation of the intervention and the contextual factors that shape this, as well as the intervention’s underlying mechanisms of action. Fidelity of intervention delivery will also be assessed across all intervention wards.

**Discussion:**

This study will establish the effectiveness and cost-effectiveness of the YCNY intervention which aims to improve patient safety and experience for older people during transitions of care. The process evaluation will generate insights about how the YCNY intervention was implemented, what elements of the intervention work and for whom, and how to optimise its implementation so that it can be delivered with high fidelity in routine service contexts.

**Trial registration:**

UK Clinical Research Network Portfolio: 44559; ISTCRN: ISRCTN17062524. Registered on 11/02/2020.

**Supplementary Information:**

The online version contains supplementary material available at 10.1186/s13063-023-07716-z.

## Background

For older people and those with complex needs, the transitional period of moving from hospital to home poses various risks [1, 2]. As many as one in five patients experience an adverse event during this time; an estimated 62% of these could be prevented or ameliorated [3]. Over the last decade, emergency readmission rates have been increasing with around 30% of all readmissions estimated to be avoidable [4–7]. Older people have the highest rates of readmissions suggesting that this group have the greatest need for support to improve transitions of care [7].

A meta-analysis of 92 randomised controlled trials (RCTs) of interventions to improve transitional care for older people observed a significant reduction in hospital readmissions at multiple time points up to 12 months post discharge [8]. The interventions tended to be highly complex, adopting multiple and variable components, and commencing and ending at different time-points. Consequently, delineating which components are the active ingredients is challenging [9–11]. There is some suggestion however that interventions which seek to enhance patient capacity to ‘reliably access and enact’ post-discharge care [12, 13] or which emphasise patient education and promote self-management [14] are most likely to be effective.

Qualitative evidence increasingly shows that patients and their carers have a central role in supporting safe care throughout the care pathway [15–17]. Patient involvement in care *during* the hospital stay (through retaining knowledge and capability to undertake usual care activities) may be a key mechanism for enhancing patients’ capacity to ‘reach-in’ to the health care system enabling them to optimise their care [18]. However, patient involvement in hospital care is not intuitive [19, 20] and is unlikely to be enacted without intervention or support. The mechanism for doing this has not been fully explored.

To address this knowledge gap, the Partners At Care Transitions (PACT) programme of research evaluates how and whether greater involvement of older patients and their families during a hospital stay can improve patient experience and safety at transitions of care. The Your Care Needs You (YCNY) intervention aims to support older people to *know more* and *do more* whilst they are in hospital. By preparing people during their hospital admission, they will be better supported to manage their health when they get home [21].

The intervention comprises both fixed and flexible components that can be tailored to the context of each participating ward — a so-called hybrid intervention [22]. This aligns with recent research which suggests that interventions should be standardised by the function of a component, rather than standardising interventions according to the specific form that they take [23]. Our early work and feasibility cRCT identified that patients were positive about the intervention; however, without encouragement from staff, patients were unlikely to do more than read the information provided [24–27]. Based on these findings, we refined the intervention and implementation strategy and we now seek to assess the effectiveness and cost-effectiveness of the YCNY intervention.

## Methods

### Study aims and objectives

To assess the effectiveness and cost-effectiveness of the YCNY intervention compared to care-as-usual in reducing the rate of unplanned hospital readmissions in patients aged 75 years and over with embedded process evaluation.

#### Objectives

1. To assess the effectiveness of the YCNY intervention at reducing the rate of unplanned hospital readmissions
2. To assess the effectiveness of the YCNY intervention at improving the quality and experience of transitions and quality of life
3. To assess the cost-effectiveness of the YCNY intervention compared to care–as-usual
4. To assess the fidelity of the intervention delivery
5. To investigate the implementation of the intervention, exploring contextual factors that affect the way the intervention is used in practice
6. To explore the mechanisms of action, specifically how YCNY is received and used by patients, carers and staff.

### Study design

A cluster RCT of the YCNY intervention versus care-as-usual in older people during the transition from hospital to home. Forty wards that routinely provide care for people aged 75 years and older, from up to 11 National Health Service (NHS) hospital Trusts in England will be randomly allocated to one of two arms: YCNY or care-as-usual (control). A process evaluation will also be conducted on a maximum of eight intervention wards to understand how the intervention is delivered, received, and used by staff and patients and how this is shaped by contextual factors.

The SPIRIT checklist [28], is available in Additional file 1.

### Setting

The cRCT will be conducted across NHS-funded, inpatient hospital wards that provide care for mostly older people and agree to participate in the trial. This may include older peoples’ medicine, intermediate care, respiratory medicine, orthopaedics, cardiology, surgical and stroke wards. Acute medical admission wards, and wards without regular medical input will be excluded.

Eligible patients will be 75 years and older, discharged to their own or a relative’s home, have stayed on a participating ward for at least one night, and be willing and able to provide informed consent. Where possible, informal carers will be recruited if patients lack capacity. Patients will be excluded if: they require an interpreter; are expected to be discharged to a nursing/care home or intermediate care/rehabilitation bed; are at end of life; live out of area; or have been admitted for psychiatric reasons (other than delirium or dementia).

### Randomisation

Wards will be randomly allocated in an equal allocation ratio (1:1) with 20 randomised to the Intervention and 20 to care-as-usual. Random allocation will be undertaken independently by the York Trials Unit (YTU) with minimisation using minimPy [29] and stratified by ward type (speciality), the percentage of patients over 75 years (split by ≤66% and >66%, based on the feasibility cRCT) and NHS trust.

### Patient population and sample size

Based on findings of similar interventions targeting readmission in a systematic review by Leppin and colleagues [12] and an underlying risk of readmissions of 18% for older patients (based on local hospital statistics), we anticipate an absolute difference in readmission at 30 days between control and intervention wards of between 4% and 6%. We therefore plan for a 4.5% reduction in readmissions at 30 days. Assuming 80% power, alpha = 0.05, intraclass correlation coefficient ) = 0.01, average cluster size = 140 (30–40 older people discharged per month from 40 wards for 5 months) and 10% attrition rate, 5440 participants are needed.

It would not be efficient to design the study to recruit and consent 5440 patients. Instead, we will use routinely collected data to explore readmission rates and include individual data collection of a nested cohort of participants within this larger sample. We will power the nested individual data collection cohort for our secondary outcome of quality of transitions. This will be measured by the Partners At Care Transitions Measure (PACT-M) [30] which produces an overall score between 0 and 67. Assuming a mean difference of 2.7 points, which equates to a reduction of around half an adverse event and a standard deviation of 9 (based on data from the feasibility cRCT), 170 patients per group are required (80% power, alpha = 0.05). Allowing for clustering this would increase to (assuming equal clusters of 25 patients and an ICC of 0.05) 374 patients per group. Allowing for 25% attrition (based on projected results from our feasibility study) we will recruit 500 patients per group (1000 total) which would require 40 clusters. We assume an ICC of 0.05 in the absence of published data indicating the most appropriate ICC for this setting and particular outcome.

### The Your Care Needs You (YCNY) intervention

#### Name of the intervention

The Your Care Needs You (YCNY) intervention was co-designed by patients, staff and researchers [27]. The intervention and implementation package were refined following a small formative evaluation [24] and a feasibility cRCT.(25;26) The structure and content of the intervention is described below using an adapted version of the TIDieR checklist [31].

#### Aims and underpinning programme theory

YCNY aims to improve the safety and experience of older patients as they transition from hospital back to their own homes. Our earlier work to model transitional care identified four key activities that patients hand over responsibility for, at the point of hospital admission, and then assume (to varying degrees) once they are discharged home [21]. These are managing their health and wellbeing, medications, daily activities, and escalating care needs.

The underpinning programme theory of YCNY posits that supporting patients to *know* and *do* more whilst they are in hospital, will help patients be better prepared for managing at home [21].

#### Intervention components

The intervention comprises three fixed patient-facing components.

- A patient booklet which makes explicit the opportunities for patients to be more actively involved in their care whilst in hospital to support a safer transition home. The booklet is structured around the four key activities and supports them to ask staff questions.
- A short patient film based on real patients’ stories from our earlier qualitative work [19]. The film brings to life, and seeks to underline, our hypothesised links between playing an active role within hospital and better outcomes after discharge.
- A patient advice sheet which supports patients to navigate care after discharge and seek help if needed. This will be tailored at the ward level prior to implementation.

In addition, ward staff will consider how they currently support patient involvement with respect to the four key activities, and what else they can do to enhance this. The actions that the teams decide to undertake (the flexible staff-facing intervention components) will not be prescribed — they will be left to vary according to staff preferences, current activities/initiatives on the ward that already address the four key activities, and their patient population.

#### Site engagement and implementation

**Fig. 1:**
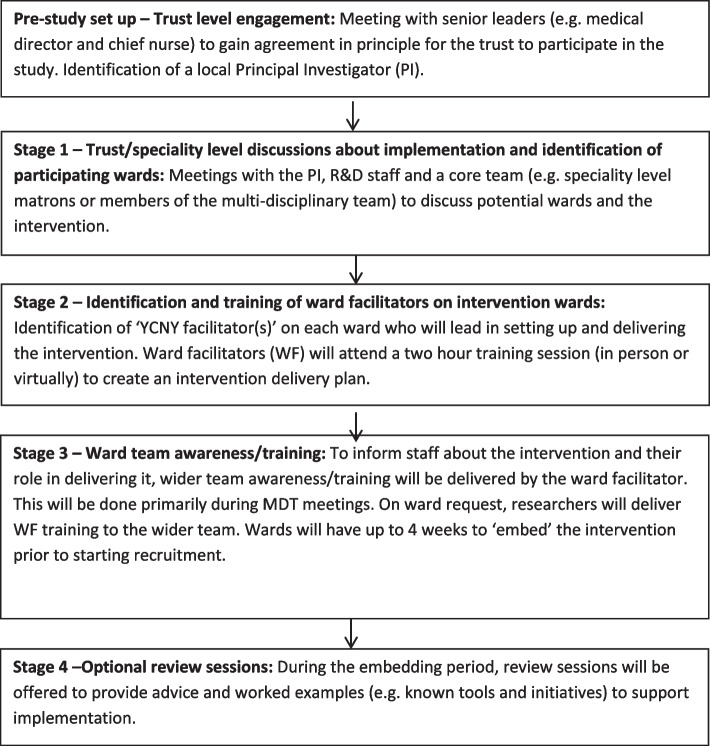
YCNY set-up and implementation steps

The intervention will be supplemented with, and supported by, an ‘implementation package’ informed by the barriers and facilitators to engagement that we have previously identified [19, 20, 24, 26] and the ‘Capabilities, Opportunities and Motivation’ Behaviour change (COM-B) model [32]. Initial engagement and set-up of the intervention will be facilitated by researchers although trusts will vary in the extent to which they want to lead on/have support for these set-up activities. The process will involve four key stages outlined in Fig. 1.

Ward facilitators will receive training and support to help them: create a plan to set up and deliver the intervention; support other activities such as creating wider staff awareness; signpost to a YCNY training and education microsite; and encourage ward staff to interact with the intervention. Specifically, ward facilitators will:

- Motivate staff to engage with the intervention by promoting it as a way to consistently support good patient involvement and communication between staff, patients and carers. Suggestions such as helping patients to write down questions and reminding them (how and when) to use the booklet, responding to resultant queries and showing it to family members will be offered. The breadth of opportunities to use the booklet lends itself to a multidisciplinary team approach so motivating all staff to engage with the intervention in various ways will be key.
- Ensure that staff feel confident to introduce and engage with the booklet and film. A short script and some prompt cards are provided to help staff explain the booklet and film to patients. Videos on the microsite also demonstrate different ways the booklet could be introduced to patients.
- Ensuring that staff, patients and carers are prompted to engage with the intervention as a whole through the provision of posters tailored at the ward level.

Beyond this, integrating YCNY into the existing roles and responsibilities of team members is a known facilitating factor in the normalisation of an intervention into routine practice [29].

#### Delivery

Ward staff will be asked to deliver the intervention to all patients who are returning back to their own homes irrespective of age for the 4- to 5-month period during which patient recruitment takes place on the ward. This is to ensure that YCNY becomes usual care on the intervention wards. In the trial, however, only those meeting our eligibility criteria (i.e. adults aged 75 years and over and returning to their own or a relative’s home) will be recruited to follow-up. Recruitment will start on completion of the intervention embedding period. Patients who consent to the trial are likely to have been exposed to the intervention, although this will not be a condition of participation.

#### Control wards

Patients on control wards will receive care-as-usual — “The wide range of care that is provided in a community whether it is adequate or not, without a normative judgement” [33]. Care-as-usual will be provided by secondary care, primary care, community and social services and will be available to both intervention and control participants.

### Recruitment and consent

Recruitment will commence up to four weeks after the embedding period. Exact start dates for recruitment will vary by Trust and ward. Recruitment will last a minimum of between four and five months on each ward.

#### Screening and identification

To recruit the nested sample of 1000 patients, all patients aged 75 years and over will be screened for eligibility. Screening logs will record the numbers of eligible patients, key reasons for ineligibility, and recruitment/refusal rates. Screening data will be used to complete a CONSORT diagram for cluster trials.

#### Approach and consent

Potential participants will be approached as soon as possible after screening. Patients will be provided with a written information sheet and researchers will verbally discuss the study in further detail. All patients will have the opportunity to ask questions. Patients (and carers if needed) will then be given as much time as they need to decide whether or not to take part. If patients wish to take part, a written (or witnessed) consent form will be completed. With participant permission, the patients’ general practitioners (GPs) will be informed of their involvement in the study. This trial does not involve collecting biological specimens for storage.

#### Patients who lack capacity

A significant proportion of patients within this older population (75 years and over) are likely to lack capacity to make decisions about their care. These patients are often more vulnerable to safety incidents and poorer experiences during transitions of care [34]. It is important that this particular patient population is included in this study to promote inclusivity in research and enhance generalisability. Capacity will be assessed during the screening process and initial approach. If patients lack capacity, attempts will be made to identify and recruit an informal carer (e.g. family member or friend) who can act as a personal consultee for the patient. A written declaration will be gained from all participating consultees. If a personal consultee cannot be identified then the patient will not be recruited into the study.

For participants who lose capacity or feel unable to complete outcome measures at follow-up, but who are otherwise happy to remain in the study, researchers will seek to identify a carer who can act as a consultee and support data collection on behalf of the patient.

#### Withdrawal

Participants and consultees are free to withdraw from the study at any point, without needing to provide a reason for their withdrawal. However, the reason for withdrawal will be recorded if provided.

During the study, follow-up data will be collected at three time points post-discharge. Researchers will make up to four attempts to contact the participant at each time point. Failure to make contact will be recorded as missing data and the participant will be contacted again at the next scheduled follow-up. Participants will only be withdrawn from the study if they or their consultee request it, the patient is deceased, or they are lost to follow-up, i.e. no longer contactable.

### Outcomes

The *primary outcome measure* (*N*=5440) is unplanned hospital readmission rates at 30 days post-discharge. It will be assessed using routine data recording the dates of all unplanned hospital readmissions up to 30 days post-discharge from the participant’s index admission.

*Secondary outcome measures* are listed below with further details available in Supplementary file 1:

- Patient At Care Transitions Measure (PACT–M) [30]
- Care Transitions Measure 3 items (CTM-3) [35]
- Questions regarding post-hospital syndrome [36]
- EuroQol 5-Dimension Health Questionnaire (5 levels) (EQ5D-5L) and Proxy EQ5D-5L [37]
- Healthcare Resource Use [38]
- Questions regarding exposure to and utility of the intervention
- Unplanned hospital readmissions at 60 and 90 days.

Permission to obtain unplanned hospital readmissions data from non-individually consented patients was provided through the United Kingdom Confidentiality Advisory Group (21/CAG/005). Patients will be able to opt out of this process through the provision of an information leaflet on the wards that details the contact details for the research team.

### Other data collected

Routinely collected data will also be used to identify length of stay of the index admission (an indicator of exposure to intervention), ward moves (an indication of potential contamination, to be gathered for consented patients only), length of stay of all unplanned hospital readmissions (for cost-effectiveness analysis), length of time to each unplanned readmission, age at index admission, gender and death within 30, 60 or 90 days after discharge from the index stay.

Data will also be collected to assess the fidelity of intervention delivery using a fidelity ‘grid’ developed as part of our trial feasibility study [26]. Fidelity will be measured across a range of areas including adherence (content, coverage, frequency, and duration) and relevant moderators such as participant (staff and patient) engagement, quality of delivery, and context [39]. A range of data collection methods will be utilised to assess the fidelity of the intervention, including ward-level observations undertaken by local research nurses, checks on intervention materials given out by wards and their usage, and discussions with ward managers and ward facilitators at the end of recruitment. Fidelity information will also be collected from patients during the follow-up questionnaires. This data will be used in a Complier Average Causal Effect analysis [40]. A total score will be produced by summing scores for the six criteria and will provide a basis for assessing the impact of non-adherence on treatment effect estimates.

### Data collection

**Fig. 2:**
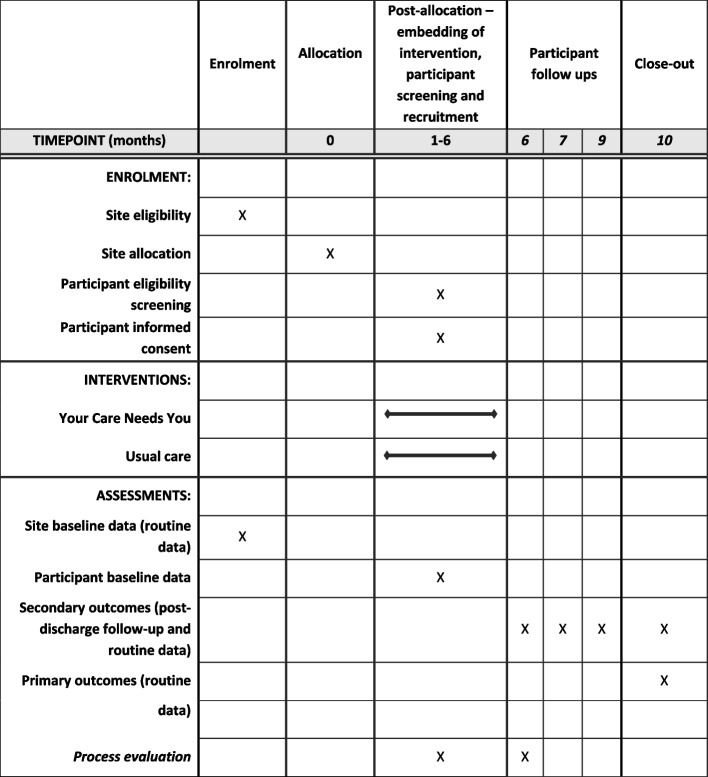
The schedule of enrolment, interventions, and assessments (as per Standard Protocol Items: Recommendations for Interventional Trials SPIRIT) [28]

The Schedule of Events table (Fig. 2) below outlines the assessments to be undertaken during this study. Assessments will either be administered by a member of the research team or self-completed with or without support e.g. through telephone assistance from a member of the research team or by recording participants’ answers for them on a postal questionnaire. Consultees will complete assessments for those who lack capacity.

### Blinding

Within this study it will not be possible to blind treatment allocation to ward or research staff who are involved in recruitment. The intervention will become usual care on the intervention wards and posters are used to communicate information about Your Care Needs You to patients, staff and visitors. Blinding of outcome assessment will be assured for the study team and data analysts except in cases where the researcher is required to conduct telephone follow-ups to obtain secondary outcome measures. In these rare cases, the researcher will have access to information in the data management system about the ward the patient was admitted to. It is possible that researchers will remember those wards that are designated as intervention wards and control wards, meaning that blinding may be compromised in these cases.

### Baseline assessments

Routinely collected ward-level baseline data will be collected for the participating wards (Table 1). In addition, once eligible participants have provided informed consent, individual-level baseline data will be collected either directly from the participant or via trust staff (see Table 1).

**Table 1:** Baseline data

**Measure**	**Description**
**Ward-level baseline data:**	
Ward-level readmission rates	Number of patients discharged by participating ward and the total number of 30-day readmissions to any ward in the hospital trust. Collected for the 12-month period of 2019 (prior to the COVID-19 pandemic). Data will be reported on a monthly basis and will be dichotomised by age (< 75 years and ≥75 years).
Average length of stay	Collected for the 12-month period of 2019 (prior to the COVID-19 pandemic). Data will be reported on a monthly basis and will be dichotomised by age (< 75 years and ≥75 years).
Age of patients	Median and mean ages of patients. Data collected for the 12-month period of 2019 (prior to the COVID-19 pandemic) and reported on a monthly basis.
**Individual level baseline data:**	
Patient demographics	Age; gender; ethnicity; first language; living/carer arrangements.
EuroQol 5-Dimension Health Questionnaire (5 levels) (EQ5D-5L) and Proxy EQ5D-5L [37]	A measure of health state (quality of life) comprising five dimensions: mobility, self-care, usual activities, pain/discomfort, and anxiety/depression. Scores can be used to facilitate the calculation of quality-adjusted life years (QALYs).
Functional Co-morbidity Index [41]	A sum of 18 self-reported comorbid conditions with a score of 0 to 18. A higher FCI score indicates greater comorbidity and is associated with impairment in physical function 1 year later,
Admission information	Date of index admission and discharge; type of index admission (planned/unplanned); reason for index admission; number of admissions in previous 12 months.

### Follow-up assessments (see the “ Outcomes” section above)

Following discharge from hospital, we will follow up recruited participants on control and intervention at three time points:

- Time 1 — post discharge: data collection will occur ideally between 5 and 17 days but up to a maximum of 21 days
- Time 2 — 30 days post discharge: data collection will occur ideally between 30 and 45 days
- Time 3 — 90 days post discharge: data collection will occur ideally between 90 and 105 days

At the point of recruitment, all participants will be advised that they will receive a questionnaire in the post and may receive a telephone call a few days later (as a reminder, to check receipt of the questionnaire or to offer support to complete). For participants who do not return questionnaires, a reminder will be sent out in the post and following this, one more reminder phone call will be made totalling up to four attempts to contact participants at each follow-up. Where contact is not made this will be recorded as missing data. Subsequent follow-ups will be initiated as planned. Researchers will not contact patients who have died since discharge.

If a patient loses capacity during the trial and therefore cannot complete the outcome measures, recruitment procedures will be followed to identify a personal consultee. Follow-up data will only be collected about participants who have lost capacity if the appropriate consent is in place. Participants will be given an unconditional £5 voucher at each follow-up time point.

### Data management, monitoring and safety reporting

Patient data will be recorded on case report forms (CRFs). Participants will be assigned a unique identification number and all data will be anonymised for analysis and reporting purposes. Electronic data (including qualitative data) and wet ink copies of the CRFs will be stored securely at the York Trials Unit (YTU) or Bradford Institute for Health Research. Data will be monitored for quality and completeness by YTU.

The trial is overseen by the Trial Management Group (TMG) comprising of the chief investigator, key co-applicants, and the operational members of the Yorkshire Quality and Safety Research group (YQSR) and YTU. An independent combined Trial Steering Committee (TSC) and Data Monitoring Committee (DMC) comprising academic, NHS England, clinical, and a patient representative meet bi-annually. Further details are available on request.

In this patient population, as death, falls, pressure ulcers, and medication issues are expected for some, these events will not be subject to expedited reporting to the main Research Ethics Committee (REC), but will be reported annually to the REC (in routine annual progress reports). There is no anticipated harm and compensation for trial participation. Quantitative monthly patient safety incident reports (via Datix) and qualitative contextual data (where required) will be gathered throughout the study. We will compare patient safety incident data in intervention wards against the same month in the previous year. Further contextual data will be sought from ward managers and local principal investigators if trends in data suggest an increase in events during the intervention period. This information will be reviewed regularly at meetings and via emailed reports from the TMG to the PMG and TSC/DMC in accordance with the Trial Monitoring plan.

### Statistical analysis

A CONSORT diagram will document the flow of wards and participants through each stage of the trial.

Readmission rates will be summarised descriptively at each time-point by treatment group and overall. The primary analysis will use a repeated-measures mixed model to compare the treatment groups. This will account for the hierarchical nature of the data by including Trust and ward as random effects and the repeated measurements from participants will be modelled by the covariance structure. The outcome will be readmission (yes/no) at 30, 60 and 90 days and the model will include important baseline covariates (e.g. minimisation factors), treatment group and time as fixed effects. An interaction term assessing whether the difference between the treatment groups changes over time will also be included in the model.

The primary analysis will compare the groups at 30 days. Secondary analyses will compare the two groups at 60 and 90 days post-discharge

Detailed statistical methods will be outlined in a separate Statistical Analysis Plan.

### Cost-effectiveness analysis

A cost-effectiveness analysis will be conducted alongside the RCT described above. The analysis will take the perspective of the NHS and Personal and Social Services, consistent with the National Institute for Care Excellence (NICE). A within-trial analysis will be conducted initially examining the costs and outcomes observed within the trial period. We will extend the time horizon if there are substantial differences between the groups in re-admission rates and/or adverse events at the final follow-up (90 days). Where extrapolation beyond one year is conducted, discounting will be applied at recommended rates (currently 3.5% per annum on costs and effects). The extrapolated analysis to a longer time horizon will be the primary analysis.

We propose to assess the cost-effectiveness of the YCNY intervention by collecting data on the costs of the intervention, utilisation of health care, and key patient outcomes.

Administered to patients or their proxy:

- The EQ5D-5L will be administered at baseline and at the three post-discharge follow-ups (T1, T2, and T3).
- The number of presentations to healthcare professionals, e.g. outpatient appointments, day case appointments, accident and unplanned attendances and use of community services.

From routine data:

- The number and duration of readmissions to the hospital trust following the index admission

From published literature:

- Unit costs of health care

To estimate the cost of the intervention:

- The costs associated with producing the booklet, and the instructions to patients document (a per-participant cost).
- The costs associated with developing the patient film (a one-off cost).
- The costs associated with staff implementing the intervention i.e. introducing the intervention and in applying the instructions to the patient’s documents. We will record which staff are involved, their grade, and, if possible, how many minutes of their time it takes (a per-participant cost).

### Embedded process evaluation

The embedded process evaluation aims to assess the fidelity of the intervention, exploring contextual factors that shape the delivery of YCNY and how it is used in practice.

Data generated will be used to interpret trial outcomes and optimise the intervention and implementation package for future use.

**Fig. 3:**
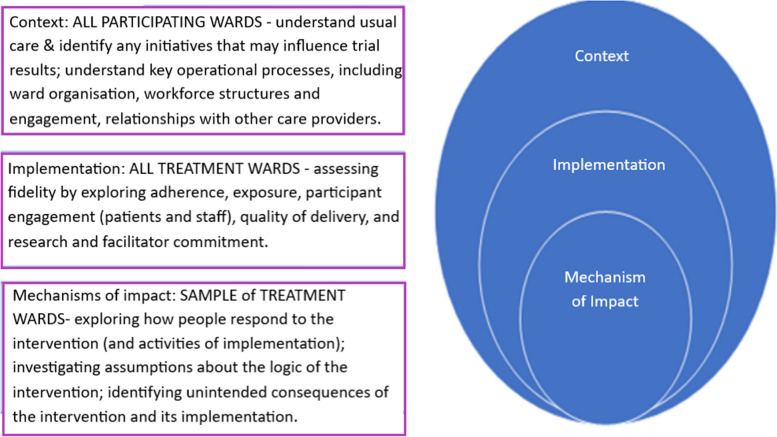
Key objectives of the YCNY process evaluation

Guidance on the design of process evaluations [42] proposes that evaluations of complex interventions should explore: context, implementation, and mechanisms of impact. In keeping with this guidance, the YCNY process evaluation has three key objectives (see Fig. 3) aiming to investigate:

1. *Context* within which YCNY is delivered (treatment wards) and factors that may impact trial outcomes (e.g. contamination)
2. *Implementation* of the intervention, specifically exploring what is delivered, how, and by whom
3. *Mechanisms of impact*, specifically how the intervention is received and used by key stakeholders, especially patients and staff

Data about site/ward set-up and early intervention delivery will be generated through structured reflections by researchers. These will be used to capture researcher perspectives on-site engagement, barriers and facilitators to intervention implementation and delivery, and other details which they feel may influence outcomes.

### Mechanisms of impact data

A more detailed ethnographic study will be conducted on approximately eight intervention wards. This will include:

- Staff experience interviews: approximately 24 semi-structured interviews will be conducted with staff involved in delivering the intervention, including the YCNY facilitators;
- Observation of care with staff and patients/carers to see how people interact with the intervention and with one another in light of the intervention as usual care;
- Semi-structured interviews with approximately 24–30 patients and their carers about their experiences of and perspectives on the YCNY intervention and the care they received whilst in hospital. We will also ask to look at and analyse YCNY booklets that patients have used.

All staff, patients and carers invited to take part in the ethnographic study will be provided with an information sheet and the opportunity to ask questions with time to consider participation. Consent will be documented for those who agree to take part.

### Data analysis within process evaluation

Data from the interviews, observations and intervention booklets will be analysed using a ‘pen portrait’ method [43]. Pen portraits are used to synthesise data across different sources. All data related to a ward will be drawn together to describe ward characteristics, how the intervention was implemented and delivered, how people (staff and patients) engaged with it, contextual factors which shaped its delivery/use, and patients’ experiences and views of it.

### Trial organisation and administration

The cRCT is being conducted as part of a five-and-a-half-year Programme Grant for Applied Health Research (RP-PG-1214-20017) funded by the National Institute for Health and Care Research. The trial is sponsored by Bradford Teaching Hospitals NHS Foundation Trust and is coordinated by YQSR at the Bradford Institute for Health Research, and YTU at the University of York.

Approvals were gained from the North East - Newcastle & North Tyneside 2 Research Ethics Committee, Confidentiality Advisory Group, and the Health Research Authority prior to starting the study (REC reference 20/NE/0020, CAG reference 21/CAG/0054). Local NHS capability and capacity approvals were granted by all participating NHS Trusts. Any amendments to the protocol will be submitted for the required regulatory approval. The study is registered on the UK Clinical Research Network Study Portfolio (44559) and the ISTCRN (ISRCTN17062524).

### Patient and public involvement

The PACT programme grant has an active patient panel who have been involved in co-designing the YCNY intervention and have advised and supported the development of study procedures and documents. Panel members were involved in the intervention iteration after the feasibility trial and gave advice upon aspects of participant recruitment and follow-up for this definitive cRCT.

### Dissemination

Findings will contribute to the ongoing progression of the PACT programme of work. They will be disseminated widely to a broad audience including academics, clinicians, healthcare managers, policy makers, patients, the public, and participants within the study. The findings will be written up for publication in peer-reviewed journals and will be presented at national and international conferences, workshops and learning events.

## Discussion

Transitions from hospital to home can be risky, particularly for older people who often have complex health and social care needs [3]. Although limited, there is some evidence that interventions that support patient involvement offer a promising way to improve transitional care outcomes [12, 13]. The PACT programme of research therefore evaluates whether supported involvement of older patients and their families in hospital improves patient experience and safety at transitions of care. Through our earlier work [21, 24, 26, 27] we have designed an intervention that supports patients to ‘know more’ and ‘do more’ during their hospital stay so that they can manage their care at home post-discharge. The present study assesses the effectiveness and cost-effectiveness of this intervention through a cRCT. As with any complex intervention various challenges are anticipated throughout the study. The particular challenges of delivering this trial at this time (whilst health services are under sustained pressure during the COVID-19 pandemic) are outlined below.

### Recruitment and follow-up

The trial feasibility study was conducted in 2019/2020, just prior to the COVID-19 pandemic. Some of the targets for the trial were refined based on the findings from the trial feasibility study. For example, our estimates for attrition at follow-up, originally estimated at 10%, were adjusted to 25% based on the trial feasibility findings. As a consequence, our recruitment target increased from 20 to 25 patient participants per ward. We do not know what the impact of the pandemic will be on patients’ desire or ability to participate in research, particularly in this older population. Furthermore, we anticipate that increasing pressure on community services is likely to impact on recruitment and follow-up rates as patients who are medically fit for discharge to home are kept in hospital (acute or intermediate care) until care packages are available. Thus, our recruitment target might be extremely challenging to meet and might still underestimate attrition. We do not, however, anticipate any particular challenges to obtaining the routine data on readmissions.

### Contamination

In our trial feasibility study, we found that only one patient moved between an intervention and control ward. This suggested that our approach to minimise contamination, through recruiting wards that were not on the same care pathway, was successful. However, we know that since the start of the pandemic ward/bay closures are more frequent and there is a chance of more patient movement, and therefore greater contamination, between intervention and control wards than was experienced during the feasibility study.

### Implementation of the intervention

We anticipate that many of the contextual challenges that impacted the delivery of the intervention during the feasibility study will exist during the cRCT. The pandemic has undoubtedly placed additional stresses on our health services that are likely to impact on the capacity of staff to deliver the intervention. We know that a sizeable percentage of nurses have experienced burnout, post-traumatic stress disorder, and compassion fatigue (to patients and to each other) leading to staff absences [44–46]. Our intervention will necessarily create some additional work for staff, however, we hope that by asking them to reflect with empathy and compassion on patient involvement they will engage with YCNY [47].

## Trial status

This article refers to protocol version 9 dated 5/7/2021. Recruitment began on 8/11/2021 with completion expected by the end of March 2023. Post-discharge data collection is due to finish in May 2023. The study end date is October 2023 to allow for post-discharge data query clarification.

First submitted 19/01/23. Due to a processing error, resubmitted 25/7/23.

### Supplementary Information

**Additional file 1: Appendix 1.** SPIRIT checklist.

## Data Availability

Requests to access the PACT data should be made to the corresponding author and will be considered on a case-by-case basis by the Chief Investigator and Trial Management Group. All data requests for quantitative data will be managed in accordance with YTU, University of York, processes and procedures.

## Abbreviations

CAG: Confidentiality Advisory Group
CONSORT: Consolidated Standards for Reporting Trials
cRCT: Cluster randomised controlled trial
CRFs: Case report forms
CSRI: Client Service Receipt Inventory
CTM: Care Transitions Measure
DMC: Data Monitoring Committee
GP: General practitioner
NHS: National Health Service
NIHR: National Institute for Health Research
PACT: Partners At Care Transitions
RCT: Randomised controlled trial
QALYs: Quality-adjusted life years
TMG: Trial Management Group
TSC: Trial Steering Committee
UK: United Kingdom
YCNY: Your Care Needs You
YQSR: Yorkshire Quality and Safety Research group
YTU: York Trials Unit
